# Editorial: Positive Psychology Studies in Education

**DOI:** 10.3389/fpsyg.2022.845199

**Published:** 2022-02-21

**Authors:** S. Abdolvahab Samavi

**Affiliations:** Department of Educational Sciences, University of Hormozgan, Bandar Abbas, Iran

**Keywords:** Editorial, positive psychology, education, hope, self-efficacy

## Introduction

While the positive psychology movement officially began two decades ago, the theories and ideas of a positive-oriented psychology are not entirely new, dating back to decades ago and even to the beginnings of psychology. Perhaps the first comment on positivist psychology came from William James, who introduced the concept of a healthy mindset years ago (Joseph and Linley, 2006). However, positive psychology—as we know it today—can be traced back to 1998; When Martin Seligman, the founder of positive psychology and then president of the American Psychological Association, introduced the concept. Seligman emphasized that psychologists should pay attention to the main missions of psychology, namely helping others to identify and nurture own potentials, and expand the definition of psychology to include positive mental health, rather than the absence of disease (Seligman, 2002). Positive psychological manifestations have emerged in many aspects of life, including treatment, parenting, marital life and education (Bradley and Hojjat, 2017; David et al., 2017; Samavi et al., 2019; Waters et al., 2021).

Education is one of the important tools of societies in socializing students and preparing them for life in the future world. The effects of positive psychology on education have been numerous (Chodkiewicz and Boyle, 2017; Shoshani and Slone, 2017). In particular, one approach in education is positive Schooling approach that supports individual care, trust, and respect for differences. In such a positive environment, teachers set an appropriate goal for each student to be interested in learning and help other students achieve the goals. This causes the hope and self-efficacy of learners to increase simultaneously (Deb, 2018).

Numerous concepts have been introduced in positive psychology related to education, the most important of which are academic hope, self-efficacy, mental wellbeing, and quality of life in school. Several studies have supported the positive role of these constructs in students' academic, motivational, and emotional outcomes (Rand, 2009; Honicke and Broadbent, 2016; Esmaeili et al., 2019). Also, other studies have pointed to the effect of positive psychological constructs on teachers' performance (Sezgin and Erdogan, 2015; Poulou et al., 2019). However, the theory of hope (Snyder, 2000), and the theory of self-efficacy (Bandura, 2006) are two positive psychological constructs that have widely influenced students' academic and motivational behaviors. The construct of self-efficacy has been considered in cognitive and motivational domains and its effect has been confirmed on academic and motivational variables. Self-efficacy refers to an individual's beliefs about his or her ability to learn or perform a behavior at an acceptable level (Maddux and Gosselin, 2012).

Academic self-efficacy refers to an individual's belief in the ability to successfully complete an academic task at a set level or to achieve an academic goal (Schunk and Pajares, 2002). Beliefs in academic self-efficacy affect task choice, effort, perseverance, sustainability, and achievement (Schunk and DiBenedetto, 2021). Also, academic self-efficacy affects motivation, learning and academic achievement (Usher and Pajares, 2006; Yusuf, 2011).

On the other hand, numerous studies have pointed to hope as a positive component that affects people's cognitive and emotional outcomes. High levels of hope have been associated with psychological wellbeing, coping with stress, adjusting to emotional distress, self-esteem, social competence, self-efficacy, and academic achievement.

In Snyder's research, hope is related to purposeful behavior and causes a person to use the skill, ability and perseverance to find a way to the goal. Hope is a positive expectation for achieving a goal that consists of two components namely, agency and path. Agent thinking is a motivational component that motivates a person to achieve a goal, while path thinking refers to choosing and finding appropriate paths to the goal (Snyder, 2000).

Hope is not only a goal-oriented cognitive process, but also an organized hierarchical system of beliefs about one's ability to engage in such a cognitive process. These beliefs are organized into three specific levels of abstraction: the general level or trait hope, domain-specific hope, and goal-specific hope (Snyder et al., 1997). People who have high levels of general hope are also hopeful in most areas of life. But in the case of students, there is usually a gap between these two levels of hope. For example, students who have high levels of hope in their lives in general may have low levels of hope in a particular field of study. In general, a comprehensive approach to understanding students' goals in education and life requires an evaluation of their hierarchy of beliefs of hope. However, since these three levels affect each other, in most cases, strengths or weaknesses are transferred from one level to another.

## Conclusion

Positive psychology, along with other areas of life, has been broadly discussed in education, and research evidence has supported many effects of this approach on learners' cognitive and emotional outcomes. It seems that in the current complex situation with the negative impact of Covid-19 Epidemic on various aspects of learning and motivation of learners, a positive psychological approach and positive interventions can be considered a good strategy for educational interventions. In the special issue related to positive psychology in education, an attempt was made to study various aspects of the positive psychology system in education, which was relatively successful. It is suggested that in order to deal more effectively with the negative consequences of Covid-19 Epidemic, positive interventions to increase the hope and self-efficacy of learners should be designed and implemented in schools. Also, due to the reopening of schools in many countries, it is necessary to design a positive educational environment in schools since it will have several consequences on the mental health of learners. Teachers can also have a strong positive impact on any student in terms of creating an environment free of fear and encouraging students to express their wants. Although the design of intervention programs based on positive psychology can be of great value, the development of a curriculum based on the concepts of positive psychology can help prepare the ground for the effectiveness of these interventions.

## Author Contributions

The author confirms being the sole contributor of this work and has approved it for publication.

## Conflict of Interest

The author declares that the research was conducted in the absence of any commercial or financial relationships that could be construed as a potential conflict of interest.

## Publisher's Note

All claims expressed in this article are solely those of the authors and do not necessarily represent those of their affiliated organizations, or those of the publisher, the editors and the reviewers. Any product that may be evaluated in this article, or claim that may be made by its manufacturer, is not guaranteed or endorsed by the publisher.
